# Measuring Hordein (Gluten) in Beer – A Comparison of ELISA and Mass Spectrometry

**DOI:** 10.1371/journal.pone.0056452

**Published:** 2013-02-28

**Authors:** Gregory J. Tanner, Michelle L. Colgrave, Malcolm J. Blundell, Hareshwar P. Goswami, Crispin A. Howitt

**Affiliations:** 1 Commonwealth Scientific and Industrial Research Organisation Plant Industry, Canberra, Australian Capital Territory, Australia; 2 Commonwealth Scientific and Industrial Research Organisation Food Futures Flagship, Riverside Corporate Park, North Ryde, New South Wales, Australia; 3 Commonwealth Scientific and Industrial Research Organisation Animal, Food and Health Sciences, St Lucia, Queensland, Australia; Tsinghua University, China

## Abstract

**Background:**

Subjects suffering from coeliac disease, gluten allergy/intolerance must adopt a lifelong avoidance of gluten. Beer contains trace levels of hordeins (gluten) which are too high to be safely consumed by most coeliacs. Accurate measurement of trace hordeins by ELISA is problematic.

**Methods:**

We have compared hordein levels in sixty beers, by sandwich ELISA, with the level determined using multiple reaction monitoring mass spectrometry (MRM-MS).

**Results:**

Hordein levels measured by ELISA varied by four orders of magnitude, from zero (for known gluten-free beers) to 47,000 µg/mL (ppm; for a wheat-based beer). Half the commercial gluten-free beers were free of hordein by MS and ELISA. Two gluten-free and two low-gluten beers had zero ELISA readings, but contained significant hordein levels (p<0.05), or near average (60–140%) hordein levels, by MS, respectively. Six beers gave false negatives, with zero ELISA readings but near average hordein content by MS. Approximately 20% of commercial beers had ELISA readings less than 1 ppm, but a near average hordein content by MS. Several barley beers also contained undeclared wheat proteins.

**Conclusions:**

ELISA results did not correlate with the relative content of hordein peptides determined by MS, with all barley based beers containing hordein. We suggest that mass spectrometry is more reliable than ELISA, as ELISA enumerates only the concentration of particular amino-acid epitopes; this may vary between different hordeins and may not be related to the absolute hordein concentration. MS quantification is undertaken using peptides that are specific and unique, enabling the quantification of individual hordein isoforms. This outlines the problem of relying solely on ELISA determination of gluten in beverages such as beer and highlights the need for the development of new sensitive and selective quantitative assay such as MS.

## Introduction

Subjects suffering from coeliac disease, gluten allergy and intolerance are advised to adopt a lifelong avoidance of gluten containing foods and beverages, such as flour, malt or beer made from barley. The typical gluten-free diet consists of food with higher GI, reduced fibre, increased fat, higher cost and poor palatability [1], [2]. Adoption of a gluten-free diet predisposes coeliacs to a higher body mass index and increased levels of obesity [3].

Gluten is a collective term for several hundred homologous, alcohol soluble, seed storage proteins in wheat (gliadins and glutenin), oats (avenins), barley (hordeins) and rye (secalins). In barley there are four protein families of hordeins: B-, C-, D- and γ-hordeins, however the B- and C-hordeins together account for over 90% of barley hordeins [4]. Isolation of hordein double-null barley lines from F2 hybrids of Risø 56 and Risø 1508 has produced an ultra-low gluten barley line (ULG 2.0) which does not accumulate B- or C-hordeins. ULG 2.0 has 3% of wild type hordein and 20-fold reduction in reactivity in T-cell assays [5]. This barley line, along with the parents, contain known but varied hordein compositions and provide a suite of grains suitable for investigating the effect of grain hordein composition on the hordein content of flour, malt, wort and beer. In an accompanying paper (Tanner this issue, this journal) we show that accurate determination of hordein requires a hordein standard, used to calibrate the ELISA reaction, identical in composition to the hordeins present in the test substance. In practice this requirement is extremely difficult to satisfy.

There is a significant research effort worldwide to further understand coeliac disease and develop alternative options for those on a gluten-free diet. Characterisation of the epitopes within gluten that are immunoreactive to coeliacs has shown that only three immunodominant peptides, derived from Ω-gliadin, C-hordein, and Ω-secalin were responsible for the immunoreactivity of the many hundred gluten proteins from wheat, barley and rye [6]. This has led to the potential for a novel peptide based therapeutic approach. Various technical solutions are also available to reduce the concentration of hordeins in beer, below the level where they affect sensitive subjects. These include selective precipitation of hordeins with tannins, or PVP [7], [8]. In addition the proteins may be hydrolysed by prolylendopeptidases [8], [9], [10], [11], [12], [13], [14]. It is not yet clear if the hydrolysed peptides remain immunogenic to coeliacs. It is more likely that hydrolysis after proline residues may not reduce the reactivity of specific glutamine resides which are the principle component of immunogenic reaction in coeliacs [15], [16]. Other food processing procedures such as transamidation of flour with microbial transglutaminase and lysine methyl ester downregulated IFN-*γ* production *in vitro* [17] and reduced the number of clinical relapses in patients challenged with transamidated bread [18]. Selection of grains with fewer immunogenic epitopes is also a possibility. Antibody guided searches have found such wheat [19], [20], [21], barley [22] and oat accessions [23]. There have also been attempts to reconstruct hexaploid bread wheats that contain a reduced number of immunogenic epitopes by using molecular approaches to select and recombine varieties [19], [24] or via the reconstruction of new hexaploid bread wheats from tetraploid and diploid progenetors that contain fewer immunogenic epitopes [25], [26], [27].

These approaches may reduce the gluten content in food, but are unlikely to completely eliminate dietary gluten. Brewing with less immunogenic grains such as oats, and gluten-free grains such as buckwheat, sorghum and millet have also been reported [28], [29], [30], [31], [32].

There remains a need for a test that can rapidly and accurately quantify gluten levels in food and beverages. The two methods approved by the WHO are antibody based tests using ELISA technology, and rely on antibodies raised against either wheat Ω-gliadin or rye secalins respectively. An advance on the standard ELISA approach using antibodies directed against another immuno-dominant peptide, the protease-resistant gliadin 33mer, have also been reported [33], [34], [35].

We show here that the hordein level in 60 commercial beers determined by sandwich ELISA varied by several orders of magnitude. These same beers were previously analysed by MS [36]. Unfortunately, there was no relationship between the total hordein content determined by optimised ELISA and the relative content of individual hordein peptides determined by mass spectrometry (MS). In practice, beers may be produced from a blend of wheat and barley varieties, so it is not always possible to identify appropriate controls. In addition proteins, including the hordeins, may be modified by hydrolysis, glycosylation (enzymatic covalent addition of a sugar) or glycation (the non-enzymatic, covalent addition of a sugar) particularly during malting. *N*-linked glycosylation occurs at the nitrogen of asparagine or arginine side chains, while *O*-linked glycosylation occurs at the hydroxyl oxygen of serine and threonone, and to a lesser extent to tyrosine and hydroxylysine or hydroyproline side chains. Glycation occurs predominantly at lysine residues, particularly during mashing [37], [38]. The addition of a sugar to a lysine residue may either: (1) mask an antibody binding epitope and thus reduce the ELISA response; or (2) it may prevent tryptic cleavage of a protein at the glycated site, interfering with the detection of a target peptide by MS. We did not find extensive evidence of glycation of the hordeins. We show in a related paper that ELISA must be calibrated with a standard with a hordein composition identical to that found in the food (Tanner et al this volume, this journal). The identification of a suitable hordein standard is difficult, and the use of a single hordein standard purified from a flour sample is a poor approximation to the hordein composition in beer. ELISA results are at best, a relative indication of the hordein levels in beer, and there is a need for a rapid MS based method. The first step, provided in Colgrave et al [36] and refined here, is the development of a relative MS based method.

## Results

### Hordein and alcohol soluble protein levels in flour

The total protein content of flour from the four test lines varied from 12.9% (w/w; or 129 mg/g) for cv Sloop to a high of 15.4% for Risø 1508. Malt contained a slightly lower range of protein concentrations ranging from a low of 9.0% for cv Sloop to a high of 12.7% from ULG 2.0, presumably because a proportion of protein reserves were mobilised during germination. Wort contained approximately 3 mg/mL of protein and beer contained from 0.7 (ULG 2.0) to 2.8 (Sloop) mg/mL protein. The average protein content of the four test beers was 1.85±0.23 mg/mL (Table S1 in Information S1).

### The pattern of proteins in flour, malt, wort and beer

**Figure 1:**
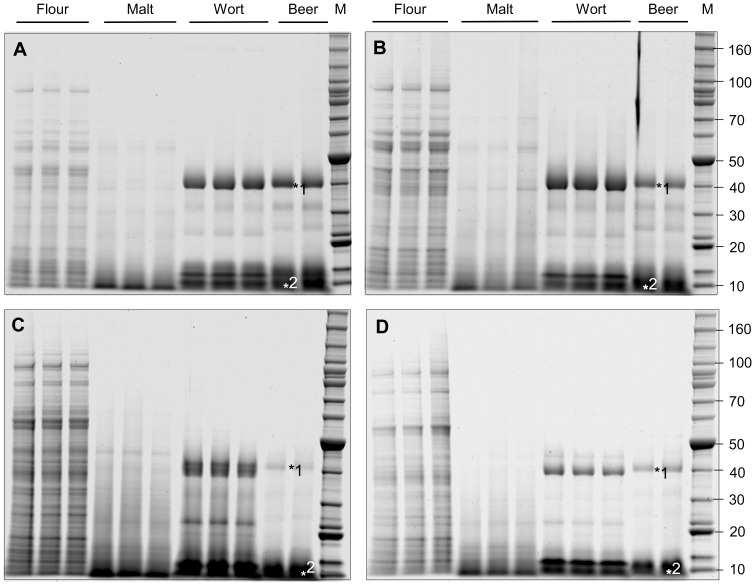
Coomassie stained protein gels (16.7 µg total protein loaded per lane) of flour, malt, wort, and beer produced from: cv Sloop (A); Risø 56 (B); Risø 1508 (C); or ULG 2.0 (D); were calibrated against Benchmark 10 kDa protein standards (Lane M, Invitrogen). The positions of serpin Z4 (*1) and LTP (_*_2) are indicated.

Staining of total protein extracts from flour samples following SDS-PAGE visualised a large number of proteins ranging from 10 kDa to 100 kDa (Fig. 1: flour). Similar analysis of malt samples identified fewer protein species with an increased intensity observed for smaller peptides (less than 10 kDa), indicating that significant proteolysis had occurred during germination and malting (Fig. 1: malt). In contrast SDS-PAGE of wort and beer, together with in-gel protein digestion and analysis, indicated that the protein composition had been dramatically enriched for two protein families, serpin Z4 at 43 kDa and LTP1 at approximately 9 kDa, accounting for the bulk of the protein in these fractions (Fig. 1; 1 & 2 respectively, Supplementary Results in Information S1, and Fig. S1 & S2 in Information S1). There were differences in the serpin Z4 proportion of the beer produced by different lines, with the most Z4 produced by beer made from cv Sloop, intermediate levels produced by Risø 56, and relatively lower proportions of serpin Z4 produced by Risø 1508 and ULG 2.0.

**Figure 2:**
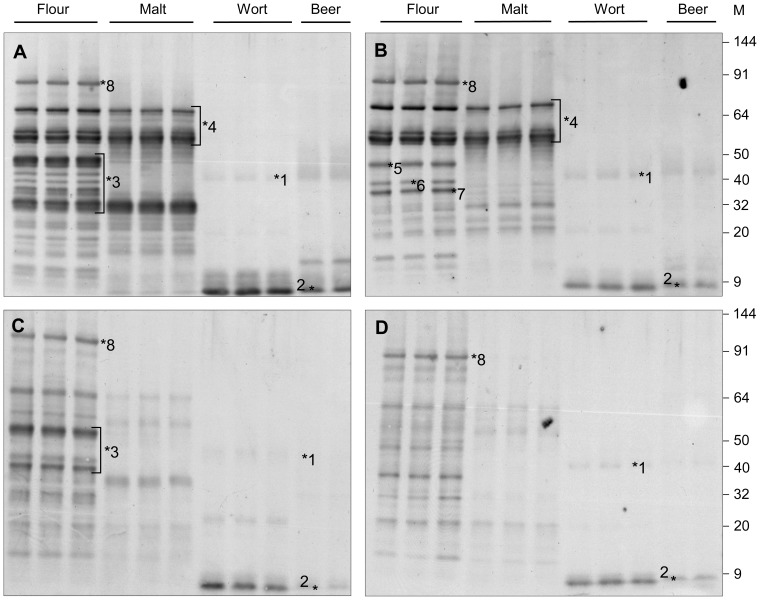
Western blot of 8.3 µg of total protein per lane visualised with 1/2000 diluted anti-gliadin-HRP from flour, malt, wort, and beer produced from: cv Sloop (A); Risø 56 (B); Risø 1508 (C); and ULG 2.0 (D). The location of known proteins was confirmed by protein sequencing of replicate gels [5]: 1, Serpin Z4; 2, LTP1; 3, B-hordeins; 4, C-hordeins; 5, γ-1-hordein; 6, γ-2-hordein; 7, γ-3-hordein; and 8, D-hordein.

Analysis of a replicate anti-gliadin western blot identified the main hordein families (Fig. 2: flour). These westerns were deliberately loaded with a high protein load to maximise the detection of hordeins in wort and beer. In all cases hordein bands in wort and beer were almost absent, indicating very low hordein levels in all wort and beer fractions (Fig. 2; wort and beer). The relative lack of hordeins in flour extracts from the hordein double-null line ULG 2.0 can be seen when anti-hordein western blots are compared with those from parental lines Risø 56, Risø 1508 and the wild type cv Sloop. Only two significant bands corresponding to known hordeins, D- and γ-3-hordein (Fig. 2D, 7 & 8), were seen in blots of total protein from ULG 2.0. At this high protein loading other proteins, in addition to hordeins, were detected presumably due to a weak homology to the epitopes detected by the antibody. This is particularly clear in the extracts of ULG 2.0 flour, where faint western bands are seen at approximately 15, 20, 32, 48, 50, and 60 kDa. Previous protein sequencing of ULG 2.0 has shown that ULG 2.0 only accumulates γ-3 hordein and D-hordein [5]. Additional bands due to serpin Z4 and LTP (Fig. 2; 1 and 2 respectively) were also seen in all wort and beer fractions, however, no hordein bands were seen in these fractions. The location of known proteins was confirmed by protein sequencing of replicate gels (Fig. 2: 1, serpin Z4; 2, LTP 1 and 2; 3, B-hordeins; 4, C-hordeins; 5, γ-1-hordein; 6, γ -2-hordein; 7, γ-3-hordein and 8, D-hordein [5].

### Response of sandwich ELISA and MS to beer

Standard curves of total hordein preparations from the test grains Sloop, Risø 56, Risø 1508 and ULG 2.0 were used to calibrate the hordein content of beers produced from these malted grains (Table 1). All other beers were diluted, so that the response was in the linear region of the H_2_O_2_ quenched Sloop total hordein standard curve, with A450 values between 0.5 and 1.5 (Fig. S3 in Information S1). The Sloop standard curve was used to calculate the hordein concentration in all other beers (Table 1).

**Table 1:** Comparison of hordein detection by MS vs ELISA.

ID	Type of beer	Grain	Avenin	B1	B3	D	γ3	ELISA^3^		MS^4^
			Hordein by Relative MS (% Sloop)^1^					Hordein (ppm)	S.E.	Wheat gluten
**Sloop**	Wild type	Barley	100.0	100.0	100.0	100.0	100.0	130	4.3	✗
**R56**	X-ray deletion	Barley	0.3	0.2	0.5	38.1	69.7	16.4	1.6	✗
**R1508**	Point mutation	Barley	1.3	0.2	0.4	12.7	65.0	0.82	0.6	✗
**ULG2.0**	Deletion/point mutation	Barley	0.1	0.1	0.4	0.6	9.0	12.7	9.7	✗
			**Hordein by Relative MS (% average)** 2							
**35**	Ale	Barley	1.1	9.8	1.0	24.6	56.2	0.05	0.00	✗
**52** *	GF	Sorghum	0.1	1.4	0.8	0.0	0.3	0.06	0.01	✗
**54**	Lager	Barley	1.6	59.9	38.2	4.0	16.4	0.06	0.00	✗
**11**	Lager	Barley	119.9	107.1	140.9	53.3	65.0	0.07	0.00	✗
**13**	Lager	Barley	51.6	50.9	36.8	92.3	124.3	0.07	0.00	✗
**53**	Light lager	Barley	117.8	180.5	305.2	133.7	151.2	0.07	0.00	✗
**57** #	Low gluten	Sorghum	49.1	327.4	2.4	104.5	62.3	0.07	0.00	✗
**58** *	GF	Barley	0.2	2.5	0.5	0.6	0.3	0.07	0.00	✗
**59** #	Low gluten	Barley	112.3	54.8	6.4	42.3	91.6	0.07	0.00	✗
**60** *	GF	Barley	0.0	2.7	1.4	0.3	0.4	0.07	0.00	✗
**50** *	GF	Sorghum	0.2	0.4	0.9	0.2	0.4	0.09	0.01	✗
**22**	Pilsner	Barley	21.7	254.7	17.2	145.3	138.6	0.10	0.00	✗
**51** *	GF	Sorghum	0.8	1.3	0.5	0.2	0.3	0.10	0.00	✗
**39**	Lager	Barley	45.7	266.1	34.5	188.7	184.3	0.15	0.01	✗
**17** *	GF	Sorghum	0.1	0.8	0.5	0.2	0.2	0.18	0.08	✗
**23**	Lager	Barley	9.0	81.2	19.4	107.3	131.4	0.25	0.00	✗
**42**	Lager	Barley	197.0	79.4	109.5	59.1	97.5	0.25	0.02	✗
**29**	Lager	Barley	170.2	144.7	171.6	109.8	139.8	0.26	0.01	✗
**20**	Lager	Barley	18.3	174.2	4.6	142.3	176.4	0.28	0.02	✗
**19**	Lager	Barley	2.4	320.4	51.1	115.4	180.3	0.29	0.01	✗
**10**	Lager	Barley	153.7	121.4	260.8	87.0	101.9	0.45	0.26	✗
**26**	Lager	Barley	0.9	206.1	229.1	162.3	143.0	0.49	0.01	✗
**28**	Lager	Barley	2.3	306.1	176.3	146.1	159.7	0.81	0.08	✗
**R1508**	Mutation	Barley	11.5	1.1	1.4	65.1	164.1	0.82	0.00	✗
**47** *	GF	Unknown	4.1	1.4	3.7	2.4	0.7	0.86	0.07	✗
**41**	Pilsner	Barley	70.0	82.7	15.0	84.4	135.4	0.88	0.01	✗
**25**	Lager	Barley	81.5	30.7	72.5	107.4	141.6	1.1	0.04	✗
**21**	Lager	Barley	130.5	141.7	55.8	60.6	91.9	1.17	0.02	✗
**38**	Ale	Barley	2.5	3.7	5.1	8.0	2.6	1.32	0.02	✗
**27**	Lager	Barley	48.8	348.7	90.8	146.0	148.9	1.53	0.06	✗
**48**	Light lager	Barley	247.8	157.6	184.5	106.1	90.2	1.82	0.03	✗
**49** *	GF	Millet	1.2	1.4	1.4	0.3	0.6	1.95	0.02	✗
**ULG2.0**	Deletion/point mutation	Barley	0.8	1.0	1.6	2.9	22.6	12.7	0.10	✗
**R56**	Deletion	Barley	2.5	1.2	2.0	194.9	175.8	16.4	1.6	✗
**36**	Ale	Barley	1.2	7.9	2.6	28.1	37.4	18.15	0.43	✗
**8**	Ale	Barley	61.1	42.2	106.0	61.6	59.9	35.8	13.1	✗
**Sloop**	Wild type	Barley	888.2	652.4	377.1	511.6	252.3	130	4.3	✗
**14**	Light	Barley	220.6	246.7	266.1	206.2	133.6	152	11	✗
**7**	Low carb. lager	Barley	195.7	136.9	218.8	152.3	125.8	191	22	✗
**24**	Lager	Barley	72.6	76.4	36.4	69.6	94.7	205	15	✗
**2**	Stout	Barley	55.2	39.7	68.8	87.1	152.2	220	26.9	✗
**12**	Lager	Barley	188.8	145.8	221.2	137.3	151.6	227	21	✗
**46**	Low alcohol lager	Barley	66.2	88.7	69.3	99.4	123.1	268	17.3	✗
**43**	Ale	Barley	116.4	6.3	154.5	63.8	67.8	289	0.35	✗
**1**	Dark lager	Barley	268.1	170.7	167.4	186.4	141.9	325	15.0	✗
**40**	Lager	Barley	126.4	74.4	77.1	130.7	182.1	330	31.0	✗
**3**	Stout	Barley	4.7	8.1	11.1	9.7	1.2	332	17.4	✗
**15**	Lager	Barley	134.6	116.3	222.9	172.1	147.1	341	11	✗
**4**	Stout	Barley	40.7	83.7	10.5	197.2	129.8	352	17.5	✗
**34**	Strong stout	Barley	21.0	11.0	5.4	45.1	78.0	464	18.7	✗
**44**	Stout	Barley	144.7	7.2	119.5	81.9	80.8	470	140	✗
**18**	Light	Barley	236.4	189.9	337.0	185.3	147.2	645	4.2	✗
**32**	Ale (wheat)	Wheat/Barley wheat	44.6	60.4	91.4	10.2	31.5	920	9.6	✓
**31**	Ale (wheat)	Wheat/Barley	75.1	25.7	161.8	71.3	85.5	4,730	310	✓
**16**	Ale (wheat)	Wheat/Barley	134.0	67.6	80.2	162.7	128.7	5,263	440	✓
**5**	Wheat	Wheat	381.0	108.6	132.7	87.9	53.6	7,700	45	✓
**37**	Stout (wheat)	Wheat/Barley	2.5	2.8	3.9	5.3	0.9	13,530	860	✓
**9**	Ale (wheat)	Wheat/Barley	207.6	44.0	158.7	124.9	71.7	16,811	2.0	✓
**33**	Wheat	Wheat	224.7	40.0	114.5	88.8	78.3	18,600	210	✓
**30**	Wheat	Wheat	21.3	4.2	31.0	18.3	14.2	20,200	590	✓
**55**	Wheat	Wheat	115.4	14.8	11.2	98.8	42.8	40,800	1,000	✓
**56**	Wheat	Wheat	109.9	26.9	16.4	118.5	77.2	42,300	530	✓
**45**	Low alcohol (wheat)	Wheat	113.2	6.1	71.9	76.6	40.7	46,500	640	✓
**6**	Wheat	Wheat	122.0	19.5	11.9	139.4	43.9	47,400	1,200	✓

The relative MS response for beer from cv Sloop, Risø 56, Risø 1508, ULG 2.0 or 60 international beers was determined by Colgrave *et al* [36], ranked by ELISA score, and reproduced here with permission.

1 & 2 : The peak area of the most intense MRM transition for each peptide was integrated and expressed here either as 1: % of cv Sloop, or 2: the average over all beers (excluding cv Sloop, Risø 56, Risø 1508, ULG 2.0, low-hordein and gluten-free lines).

3 : All ELISA readings were calibrated against a standard curve made from total hordeins purified from cv Sloop, except for beers made from hordein deletion lines Risø 56, Risø 1508, and ULG 2.0 which were calibrated against standard curves made from hordeins purified from the respective grains. ELISA readings less than 0.10 ppm were not significantly different to zero.

**Figure 3:**
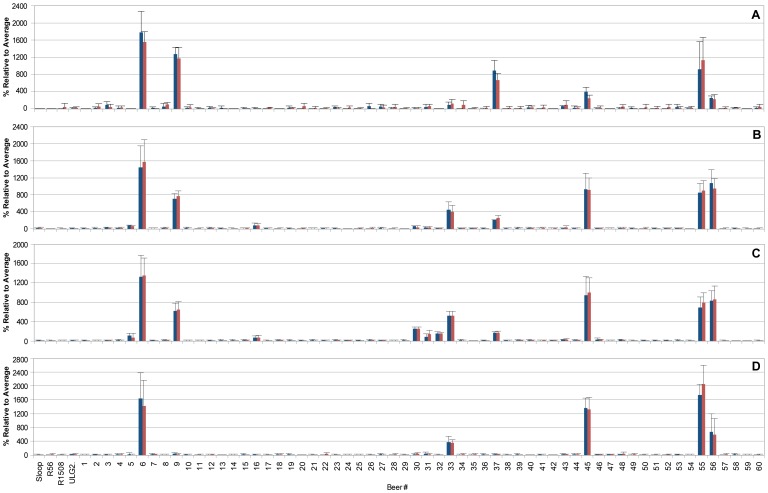
Scheduled multiple reaction monitoring (MRM) mass spectrometry revealed the presence of wheat gluten proteins in beers 5, 6, 9, 16, 30–33, 45, 55 and 56. (A) α-gliadin peptide pQQILQQQLIPCR; (B) α-gliadin peptide VPVPQLQPQNPSQQQPQEQVPL; (C) high molecular weight glutenin peptide IFWGIPALLK; and (D) low molecular weight glutenin peptide SIVLQEQQQVR. Three MRM transitions were monitored for each peptide. The summed peak areas ± S.D (n = 3) for the two most intense MRM transitions are shown, the third MRM transition was used as a qualifier (confirmation of peptide identity).

4 : MS analysis of beers after tryptic digestion revealed the presence (✓) or absence (✗) of wheat proteins: α-gliadin, γ-gliadin and low- and high- molecular weight glutenins indicative of the beer containing wheat (see Fig. 3).

* Gluten-free. The small peak area by MS seen for the gluten-free beers was due to integration of baseline noise. Values <5% were not significantly different to blank samples.

# Low gluten claim <20 ppm for this beer.

Our ELISA results show that cv Sloop beer had a higher hordein level than expected at 130 ppm (Table 1), as the hordein concentration in barley beers has been reported to vary from 19–45 ppm [39]. Beer from Risø 56 had an intermediate hordein level of 16.4 ppm (12.6% of Sloop), while ULG 2.0 and Risø 1508 beers were lower at 12.7 and 0.8 ppm respectively (9.7% and 0.3% respectively of Sloop) when measured by Elisa.

Sandwich ELISA results for all beers varied from zero (for four known gluten-free beers) to concentrations of 40,800–46,500 ppm for three wheat beers. The mean ±SE hordein content of all beers was 5,400±1,760 ppm. The large range meant that the median value of all beers was only 198 ppm. The beers with the highest hordein level by ELISA were all wheat beers (Table 1: beers 6, 30, 33, 45, 55, 56) with a mean hordein ± S.E. content of 31,900±5,000 ppm with a median value of 40,800 ppm. The beers with a zero hordein level by ELISA included beers labelled as gluten-free (Table 1: beers 17, 47, 49, 50, 51, 52, 58, 60) as well as some that were brewed using barley (beers 11, 13, 22, 35, 53, 54). The mean hordein content of all gluten-free beers, excepting beers 47 and 49, was 0.08±0.005 ppm (median 0.15 ppm), identical to the (no addition) blank reading. Two designated gluten-free beers (47 and 49) had small, but significant ELISA readings (p<0.05) and MS analysis revealed the presence of avenin like-A protein, B3- and D-hordein (>1% of the average of all beers), most likely indicating contamination by barley. Two beers with a low gluten claim (beers 57 and 59) had a zero ELISA reading, but MS revealed they contained substantial levels of hordeins with at least one peptide with a value ∼100% of the average hordein content for all non-zero beers (Table 2).

**Table 2:** Relative hordein peptide composition of designated low gluten and gluten-free beers.

Beer ID	Type	Avenin	B1	B3	D	γ3	ELISA^2^ (ppm)	
		Hordein by Relative MS (% average)^1^					Mean	S.E.
17	GF	0.1^#^	0.8^#^	0.5^#^	0.2^#^	0.2^#^	0.18^#^	0.08
47	GF	4.1^*^	1.4^*^	3.7^*^	2.4^*^	0.7^*^	0.86^*^	0.07
49	GF	1.2^*^	1.4^#^	1.4^#^	0.3^#^	0.6^#^	1.95^*^	0.02
50	GF	0.2^#^	0.4^#^	0.9^#^	0.2^#^	0.4^#^	0.09^#^	0.01
51	GF	0.8^#^	1.3^#^	0.5^#^	0.2^#^	0.3^#^	0.10^#^	0.00
52	GF	0.1^#^	1.4^#^	0.8^#^	0.0^#^	0.3^#^	0.06^#^	0.01
57	Low gluten	49.1^†^	327.4^†^	2.4^*^	104.5^†^	62.3^†^	0.07^#^	0.00
58	GF	0.2^#^	2.5^#^	0.5^#^	0.6^#^	0.3^#^	0.07^#^	0.00
59	Low gluten	112.3^‡^	54.8^‡^	6.4^*^	42.3^‡^	91.6^‡^	0.07^#^	0.00
60	GF	0.0^#^	2.7^#^	1.4^#^	0.3^#^	0.4^#^	0.07^#^	0.00
Average^3^		0.23^#^	1.52^#^	0.77^#^	0.25^#^	0.32^#^	0.08^#^	0.01

The mean and S.E. for the ELISA determination are shown along with the individual hordein peptide content by MS, determined for that beer.

Within each column, the hordein content of beers with the same symbol were not significantly different by ANOVA (p<0.05).

1: All of the peptides except γ3 contained at least one zero, indicating small (but non-missing) observed concentrations. To account for the variance, the mean MS peak areas, relative to cv Sloop were transformed with log10(1+concentration) and a one way ANOVA showed log transformed data for all peptides in beers 47 was significantly higher than the gluten-free beers 17, 50, 51, 52, 58 and 60 at the 5% level. The transformed data for beers 57 and 59 were significantly higher than all other beers. The transformed data for avenin in beer 49 was significantly higher than that for the gluten-free beers (Table S4).

2: The mean ELISA level was log transformed to reduce the variance. One way ANOVA showed log transformed data for both beers 47 and 49 were significantly higher than the reading for the remaining beers (Table S3).

3: Average of GF lines (excepting 47, 49, 57, and 59).

Commercial beers 11, 13, 22, 35, 53, and 54 did not react with the ELISA Systems kit (mean ELISA reading 0.1±0.01 ppm) suggesting that the hordein content of these beers was zero. Subsequent MS analysis showed these beers contained substantial hordein levels with values for at least one peptide greater than 50% of the average (Table 3). Thus we conclude that these beers generated false negatives by ELISA. The failure of ELISA to detect hordein in these beers may be explained by protein degradation as untargeted MS analysis revealed up to a 14-fold increase in hordein peptide fragments in the sub-10 kDa filtrate from undigested beers (data not shown). Beers 11, 13, 53 and 54 showed the highest levels of these relatively small (10–12 amino acid) hordein remnants.

**Table 3:** Relative hordein peptide composition of apparently zero hordein beers (11, 13, 22, 35, 53 and 54).

	Avenin	B1	B3	D	γ3	ELISA (ppm)
	Hordein by Relative MS (% average)					
Mean	52.3	110.5	89.9	75.5	92.0	0.10
SE	22.4	37.4	47.5	23.6	21.9	0.01
Median	36.7	83.5	37.5	72.8	94.7	0.10

The mean, S.E. and median hordein composition was determined by using a representative peptide from each hordein family.

In addition, there was another group of ten apparently low hordein beers (10, 19, 20, 23, 26, 28, 29, 39, 41 and 42) that had a very low ELISA reading of <1 ppm (mean 0.38±0.08, median 0.28 ppm), but a significant MS level with several hordein peptide values close to the average (Table 4). The mean hordein content by MS for avenin-like protein, B1-, B3-, D- and γ-hordein were 67, 178, 107, 120 and 145% respectively of the level relative to the average hordein content. These beers represent a large group where the ELISA reaction was suppressed.

**Table 4:** Relative hordein peptide composition of apparently low (<1 ppm) hordein beers (10, 19, 20, 23, 26, 28, 29, 39, 41, and 42).

	Avenin	B1	B3	D	γ3	ELISA (ppm)
	Hordein by Relative MS (% average)					
Mean	67.0	178.2	107.2	120.2	145.0	0.38
SE	24.5	29.4	30.3	12.5	9.7	0.08
Median	32.0	159.5	80.3	112.6	141.4	0.28

The mean, S.E. and median hordein composition was determined by using a representative peptide from each hordein family.

Stouts had similar hordein content by ELISA (mean 360±33 ppm, median 342 ppm, excluding stout 37), but highly variable content by MS (Table 5). Beers 1, 2, 3, and 4 of Table 1 were good examples of the variation encountered. All had moderate hordein levels by ELISA (mean 307±26 ppm, median 328 ppm), however, for dark lager 1, the hordein peptides measured by MS varied from 140–270% of the average hordein content of all beers (Table 1). This contrasts markedly with stout 3, which had very low hordeins by MS, of approximately 10% of the average hordein content. Stouts 2 and 4 had relative hordeins by MS which varied from 40 to 152% of the average.

**Table 5:** Relative hordein peptide composition of stouts (beers 1, 2, 3, 4, 34, 44).

Stout	Avenin	B1	B3	D	γ3	ELISA (ppm)
	Hordein by Relative MS (% average)					
Mean	89.1	53.4	63.8	101.2	97.3	360.5
SE	41.0	26.4	27.6	30.9	23.1	32.6
Median	48.0	25.4	40.0	84.5	105.3	342

The mean, S.E. and median hordein composition of stout beers was determined by using a representative peptide from each hordein family.

Lagers were the largest group accounting for 39% of all beers assayed. In general they had lower but highly variable hordein levels with a mean ELISA content of 62.7±25 ppm (median 0.5 ppm, with a range of 0.067–341 ppm). The mean hordein content by MS for avenin like-A protein, B1-, B3-, D- and gamma-hordein were 90, 149, 118, 110 and 129% respectively of the level relative to the average hordein content (Table 6).

**Table 6:** Relative hordein peptide composition of lagers (10, 11, 12, 13, 15, 19, 20, 21, 23, 24, 25, 26, 27, 28, 29, 39, 40, 42, 46, 48, 53, 54).

Lager	Avenin	B1	B3	D	γ3	ELISA (ppm)
	Hordein by Relative MS (% average)^1^					
Mean	90.3	149.0	118.6	110.5	129.2	62.7
SE	15.6	19.3	18.9	9.3	8.9	25.1
Median	77.1	131.6	84.0	108.6	140.7	0.47*

The mean, S.E. and median hordein composition of lager beers was determined by using a representative peptide from each hordein family.

* The median value was distorted by several low readings. In this case the range is reported (0.067–341).

Ales were highly variable compared to cv Sloop, when the hordein content was measured by ELISA (mean 3,100±1,700 ppm; median 289 ppm), however, the mean hordein content by MS was less variable at about 70% of the average hordein content, e.g. means for avenin like-A protein, B1-, B3-, D- and gamma-hordeins were 72, 30, 85, 62 and 60% respectively of the level relative to the average hordein content (Table 7).

**Table 7:** Relative hordein peptide composition of ales (8, 9, 16, 31, 32, 35, 36, 38, 43).

Ale	Avenin	B1	B3	D	γ3	ELISA (ppm)
	Hordein by Relative MS (% average)^1^					
Mean	71.5	29.7	84.6	61.7	60.1	3120
SE	23.6	8.2	22.6	17.6	11.9	1740
median	61.1	25.7	91.4	61.6	59.9	289

The mean, S.E. and median hordein composition of ales was determined by using a representative peptide from each hordein family.

### Comparison of ELISA and MS determinations

The ELISA results did not correlate with the relative content of individual hordein peptides as determined by MS (Fig. S4 in Information S1). The relative concentrations of the individual proteins (avenin, B1-hordein, B3-hordein, D-hordein or γ-3-hordein) determined by MS did not correlate with the total hordein content determined by ELISA with r^2^ values for the lines of best fit less than 0.06. Nor was there correlation between the ELISA reading and the summed relative hordein concentrations for each beer. This was due, in part, to the fact that the MS analysis only considered barley proteins. Several of the beers examined in this study contained substantial amounts of glutenin and gliadin (wheat gluten).

Given the lack of correlation, we sought to find an explanation and to this end, we looked for possible post-translational modifications that would alter the ability of MS to accurately detect and quantify the hordein proteins. Tryptic digestion of wild-type (Sloop), the three deletion mutants and 60 commercial beers was undertaken and analysed by LC-MS/MS in a non-targeted manner. Typical peptide modifications, such as oxidation of methionine, were detected, however, this type of modification did not affect the outcome of the MS assay as the MRM-MS method used in the targeted approach incorporated two versions (non-oxidised and oxidised) for all Met-containing peptides. As glycosylation and glycation were expected, we also looked for the presence of these modifications. We found limited peptide evidence for glycation of lysine residues. Evidence of glycation was restricted to peptides from abundant proteins including LTP1 (Uniprot accession: P07597), LTP2 (P20145), α-amylase trypsin inhibitor CMb (P32936) and in a single peptide from D-hordein (Q84LE9). Where glycated lysine residues were present, the peptide was not cleaved by trypsin. The single instance of a glycated peptide (VAK*AQQLAAQLPAMCR) in D-hordein did not affect the MS results as an alternate tryptic peptide was selected for quantification.

### Detection of wheat peptides in beer by MS

Examination of the complete lists of proteins identified, revealed that a number of the commercial beers not labelled as containing wheat did indeed contain traces of wheat gluten proteins (beers 9, 16, 31, 32 (all ales) and 37 (stout)). For example, beer 9 (an Australian ale) yielded α-gliadin (Q9M4L6) as the primary protein identification along with γ-gliadins (Q9M4L5, Q94G94) and a low molecular weight glutenin (Q75ZV8) as lesser abundant protein identifications. Further, a number of defensins, α-amylase trypsin inhibitors and non-specific lipid transfer proteins from wheat were also identified. A similar suite of proteins were identified in beers 5, 6, 30, 33, 45, 55 and 56, all declared wheat beers. The average ELISA measurement of non-wheat containing beers was 103.3±24.2 ppm. All of the beers found to contain wheat by MS gave ELISA values well in excess of 500 ppm with nine of the eleven samples yielding ELISA values >4,000 ppm.

Subsequently, we performed MRM analysis of the beers to look for the presence of peptides that mapped to known wheat gluten proteins (α-gliadin, Q9M4L6; low molecular weight glutenin, Q75ZV8; and high molecular weight glutenin, P10388). Using this more sensitive MS assay, wheat peptides were detected in beers 5, 6, 9, 16, 30, 31, 32, 33, 37, 45, 55 and 56 with at least two wheat peptides detected in each beer (Fig. 3).

The identification of wheat proteins in a number of the beers necessitated further MS analysis to differentiate between wheat and barley gluten. Each peptide candidate was interrogated by BLAST searching on the Uniprot and NCBI servers to check for species specificity. In the case of the avenin-like A protein (Uniprot accession F2EGD5), only a single peptide was detectable in beer under the experimental conditions and this peptide was found to be present in proteins from wheat (*Triticum*), goatgrass (*Aegilops*) and barley (*Hordeum*). In the case of D-hordein (Uniprot accession Q84LE9), a suite of tryptic peptides were detected. MRM analysis was performed on the test and commercial beers using peptides derived from a tryptic digestion of D-hordein that were either: (a) unique to barley gluten (D-hordein, Uniprot accession Q84LE9); or (b) common to barley and wheat gluten (HMW-glutenin, Uniprot accession P10387). Table S2 in Information S1 lists the peptides that were investigated. The peak areas (relative to average) for 3 MRM transitions per peptide for the common (ELQESSLEACR) and unique (QYEQQTEVPSK) D-hordein derived peptides are shown in Fig. S5 in Information S1. In contrast to the non-wheat beers, beers containing wheat (5, 6, 9, 16, 30, 31, 33, 45, 55 and 56) showed an increased proportion of the common peptide.

## Discussion

Beer is a solution containing protein at approximately 1 mg/mL. Two highly modified protein families arising from glycated forms of LTP [40], [41] and serpin Z4 constitute the majority, while trace amounts of other proteins including the hordeins represent the minority. Although hordeins appear at trace levels, the levels are still too high for coeliacs and gluten intolerants to safely consume. The dominant serpin Z4 and LTP proteins together largely determine the important foaming properties of the final beer [42]. Extracts of wort and beer were dramatically enriched for serpin Z4 and LTP when compared to extracts of flour and malt. This enrichment corresponds to a loss of other water soluble proteins precipitated during boiling of the wort, the so called “hot-break” formed during the brewing process. Comparison of total protein extracts from flour with those obtained from malt showed considerable evidence of proteolysis during germination.

Comparison of total protein extracts by anti-hordein western blot showed that the hordein proteins were vastly under-represented in wort and beer. This indicates that the bulk of alcohol soluble hordein proteins did not dissolve during mashing and brewing. In western blots at high protein loads, additional bands were observed which corresponded to non-hordein proteins such as serpin Z4 and LTP 1 & 2. This reflects a limited affinity of the anti-hordein antibody towards these proteins and urges caution in interpreting antibody based results such as western blots or ELISA analysis at high protein loads.

In this study, we undertook a re-examination of the 60 beers using untargeted MS analysis on an instrument with higher sensitivity and resolution compared to that used in our previously published study [36]. The aim was to generate a proteomic profile of the beers and look for post-translational modifications and/or unusual cleavage events that might explain the lack of correlation with the ELISA results. This analysis confirmed the presence of proteins such as serpin-Z4, LTP1, α-amylase trypsin inhibitors, defensins, heat shock proteins and the hordeins, principally B-, γ-, and D-hordein. Traces of C-hordeins (Q41210, P17991 and Q40053) were also detected in undigested beer; the numbers of unique C-hordein peptides identified at 95% confidence from the above three accessions were 6, 3 and 2 respectively. In the previous study reported by our lab (using an instrument with lower sensitivity and resolution), we identified two of the above C-hordein proteins (P17991 and Q40053) [36]. The trace C-hordein peptides were random fragments produced during the malting and brewing process and were difficult to quantify due to the random nature (non-tryptic) of the cleavages that produced the peptides.

The non-targeted MS analysis of beer peptides revealed evidence of glycation occurring in at least four peptides. Glycation of the lysine preceding one of the target D-hordein peptides prevented complete proteolysis by trypsin and resulted in a decreased intensity (<20% loss) of this peptide in the MS analysis. However, glycation was only detected in a single hordein peptide. This peptide was not used for the relative quantification by MS. This finding further demonstrates the importance of prototypic peptide selection in MRM-MS analyses.

The value of MRM analysis of gluten lies in the ability of this method to not only to detect sub-clinical levels of gluten peptides, but also to interrogate the peptide sequence and confirm the identity of the parent protein. Other techniques such as HPLC and SDS-PAGE rely on comparison of migration patterns with those of standard proteins. Determination of gluten using ELISA relies on the use of a calibration standard, invariably a commercial preparation of wheat gliadin. We show in an accompanying paper (Tanner et al this volume, this journal) that accurate ELISA determination of gluten requires a standard with a gluten composition comparable to that of the food being measured. The use of a single wheat gliadin standard is unsuitable for the accurate determination of gluten from cereals which consists of a complex mix of proteins that have differing reactivity's with the antibodies used. Individual gluten proteins may be purified with some difficulty from the many hundred gluten proteins present in unprocessed grain flour using FPLC [5], however, this technique remains beyond the means of routine food laboratory analysis. The purification of a given gluten protein from processed products such as beer is complicated by the low abundance of gluten proteins, as well as modifications to the primary structure such as glycation, glycosylation and partial or complete proteolysis. These factors combine to make isolation and purification of a suitable gluten standard suitable for a variety of processed foods, impractical.

The accurate determination of the gluten level is crucial for the determination of gluten-free status of foods required by regulatory bodies. The WHO body, the *Codex Alimentarius* is the responsible body for food regulations in the EEC, and sets a limit for gluten in gluten-free food as less than 20 ppm. Food legislation in Australia and New Zealand is set by Food Standards Australia and New Zealand (FSANZ). Similar regulatory bodies in the United States (USDA), in Canada (Health Canada) also follow the international legislation set by the *Codex*. FSANZ requires food to have a gluten limit of less than 20 ppm to qualify for gluten-free labelling. In addition FSANZ requires that gluten-free food must contain no detectable gluten – this was once a conservative, “catch all” clause, however as detection limits have been lowered, particularly by MS based methods, well below the clinical threshold, clauses such as this are no longer practical.

Two ELISA methods have been ring tested and approved for determination of gluten-free status of food and beverages. These methods use either the Mendez R5 antibody [43] or the Skerritt antibody [44].

In the absence of a “universal” hordein standard we analysed the hordein content of beer samples using ELISA (calibrated against a total hordein preparation from cv Sloop) or by MS. In the absence of a suitable standard to calibrate the MS methods we compared the individual content of five prolamins (avenin like protein, and B1-, B3-, D- and γ-3-hordein) in each beer, to the level for each prolamin averaged over all beers (omitting cv Sloop, Risø 56, Risø 1508, ULG 2.0, low-hordein and gluten-free lines). The conversion of the MRM methodology from relative quantification to an absolute measurement of hordein is an area of active research within our laboratory.

The comparison of hordein content obtained by ELISA with the relative concentration of hordeins obtained by MS revealed several significant differences:

- Underestimation: Two declared gluten-free beers had zero ELISA readings, but MS indicated they contained a low level of hordeins between 1–4% of the average for all beers. Ten beers had very low ELISA readings of less than 1 ppm, but had several hordein peptides close to the overall average by MS.
- False negatives: Two low gluten barley beers had zero level by ELISA, but near average hordein level by MS. Six other beers also had zero ELISA readings, but near average hordein by MS.

This analysis shows that ELISA determination, while convenient, is no longer suitable for measurement of gluten in beverages. The MS analysis showed that all beers tested that were derived from barley contained hordein (gluten). Testing using ELISA determination should eventually be replaced with suitable MS based methods. The MS equipment required to carry out such analysis is now not uncommon and increasingly available to the food industry.

## Methods

### Plant material

Barley line cv Sloop (wild type) was obtained from the Australian Winter Cereals Collection (Tamworth, Australia). Single hordein null lines, Risø 56 (accumulating no B-hordeins due to a X-ray induced chromosomal deletion) and Risø 1508 (an ethyleneimine induced point mutation in the *lys 3a* gene which prevents accumulation of C-hordeins and decreased D- and B- hordeins) [45], [46] were obtained from the Nordic Germplasm Bank (Alnarp, Sweden), intercrossed and F2 progeny selected that lacked B- and C-hordeins. This hordein double-null seed was refined by single seed descent to produce an F6 line, Ultra Low Gluten Barley (ULG 2.0). The stock of single and double hordein-null lines was increased in the field, harvested and malts and beers prepared as described (Tanner et al this volume, this journal and [36]).

### Protein extraction

Total protein extracts were prepared by homogenising triplicate 20 mg samples of wholemeal flour in 1 mL freshly made solvent containing 8 M urea, 1% (w/v) DTT, and 20 mM triethylamine-HCl, all adjusted to pH 6 at 4°C (Urea/DTT), in a Bio101 bead beater (Savant) with 0.1 g of 0.1 mm dia. glass beads (Daintree Scientific, Tasmania), a ¼ inch ceramic bead (Bio101 systems, MP Biomedicals, California) for 30 s at speed 4, taking care not to overheat the solution. Extracts were centrifuged at 13,000 g av, and the supernatant taken for analysis.

### One dimensional SDS polyacrylamide gel electrophoresis (SDS-PAGE)

Up to 10 µL of alcohol soluble or total protein solution was diluted to a final volume of 30 µL with a solution containing 8 M urea, 2% (w/v) SDS, 62.5 mM Tris-HCl (pH 6.8), 0.01% (w/v) bromophenol blue containing 65 mM fresh DTT (Urea/SDS), left to reduce at RT for 30 min, applied to NuPage 4–12% Bis-Tris acrylamide gel (Invitrogen), calibrated with 10 kDa protein ladder (Invitrogen) and electrophoresed at 200 V for 60 min. Wort and beer solutions were lyophilised, dissolved in Urea/SDS and an aliquot containing the required amount of protein electrophoresed. Lyophilisation and dissolution in Urea/SDS produced an improved protein banding pattern compared to dilution (Fig. S6 in Information S1). Gels were fixed in 40% (v/v) methanol, 10% (v/v) acetic acid, washed in distilled water, and proteins stained in 0.006% (w/v) colloidal Commassie G250, and destained in water overnight.

### Western blotting

SDS-PAGE was carried out as above and protein gels were blotted without delay to a nitrocellulose membrane at 20 V for 7 min (iBlot, Invitrogen) and the membrane stained in a solution containing 0.2% (w/v) Ponceau-S (Sigma), 3% (w/v) trichloroacetic acid, and 3% (w/v) 5-sulphosalicylic acid. The image was scanned on an Image Scanner III (GE Healthcare) using Labscan software (GE Healthcare), the membrane destained in water, and blocked in 5% (w/v) skim milk powder in 1% (w/v) Tween in PBST for 2 h at RT. Blots were interrogated with commercial polyclonal anti-gliadin-HRP antibody conjugate (Sigma), at 1/1000 dilution in PBST, developed in ECL reagent (GE Health Care), exposed to Hyperfilm (GE Health Care). Images were developed and scanned as above. Where indicated, the membranes were then stripped in 8 M urea, 50 mM Na phosphate buffer, 20 mM DTT, all adjusted to pH 6, for 1 h at RT, rinsed, re-blocked and probed with a second antibody pair: rabbit polyclonal anti-serpin Z4, diluted at 1/2000, for 1 h followed by 1/2000 diluted donkey anti-rabbit-HRP (GE Healthcare) for 30 min and developed as above. This procedure was repeated for the third antibody pair: rabbit anti-LTP1, followed by 1/2000 diluted donkey anti-rabbit-HRP (GE Healthcare). Stripped membranes were checked for lack of residual signal before applying the next antibody. The preparation of anti-serpin Z4 and anti–LTP1 is discussed in Supplementary Methods in Information S1.

### ELISA analysis

Two ELISA methods have been ring tested and approved for determination of gluten-free status of food and beverages by the WHO body the *Codex Alimentarius*. We used a sandwich assay kit based on one of the approved methods using the Skerrit antibody, to determine hordein contents of international and local beers (#ESGLI-48, ELISA Systems, Windsor, Queensland). Two bottles of each selected international and Australian beer were purchased from local liqueur merchants and degassed. Hordein was determined in triplicate aliquots from each bottle of test and commercial beer, diluted and calibrated against a 0.2 mM excess H_2_O_2_ quenched standard curve of total Sloop hordeins prepared, thawed and diluted as described (Tanner, this volume, this journal; Table 1, Fig. S3 in Information S1). Beers made from hordein deletion lines Risø 56, Risø 1508, and ULG 2.0 were calibrated against standard curves made from hordeins purified from the respective grains as described (Tanner et al this volume, this journal). Depending on the hordein level, individual beers required a final dilution which varied from 1/2 to 1/50,000. The beers were first diluted with at least an equal volume of Urea/DTT followed by dilution with sufficient ELISA Systems diluent containing 0.2 mM H_2_O_2_ (ED buffer) to keep the final urea and reduced DTT concentrations below 100 mM and 0.2 mM respectively, so that these compounds did not interfere with the ELISA assay (Tanner et al this volume, this journal).

### Protein concentration

The protein concentration was determined by the method of Bradford [47].

### Comparison of ELISA and mass spectrometry (MS) results

ELISA yielded hordein and/or gluten values as an absolute value (in ppm) and did not distinguish different forms of hordein/gluten. The scheduled multiple reaction monitoring (MRM) MS approach described here is currently limited to relative quantification and all values were expressed as a percentage of the average content. The development of the MRM technique is described in Colgrave et al [36] and results are reproduced here with permission. The average beer content was determined by taking the mean peak area of all ‘normal’ commercial beers, *i.e.* those that were not expected to be gluten-free or low in gluten (thus excluding beers 17, 47, 49–52, 57–60). The MRM technology was able to distinguish individual protein isoforms allowing the peak area to be determined for peptides that were representative of the most abundant hordein protein in the classes: avenin, B1-, B3-, D- and γ3-hordein. The percentage was calculated by taking the peak area of the peptide of interest and dividing this by the mean (as described above) and was expressed as a percentage. Further, the values reported in Table 1 are the mean of two independent measurements (two bottles of beer, two technical replicates of each).

### Identification and relative quantification of wheat gluten proteins

Tryptic peptides were chromatographically resolved using a Shimadzu Prominence LC20 HPLC system with a C18 Vydac column (75 µm×15 cm, 300 Å, 5 µm). Duplicate tryptic digests from each different bottle were acidified in 0.1% (v/v) formic acid and 10 µL injected. A linear gradient at a flowrate of 300 nL/min, from 2–40% solvent B over 6.8 min was utilised where solvent A was 0.1% (v/v) formic acid and solvent B was 0.1% (v/v) formic acid in 90% (v/v) acetonitrile. The eluate from the HPLC system was directly coupled to the nanoelectrospray ionisation source of the TripleTOF™ 5600 system (AB/Sciex, Foster City, CA, USA). Data were acquired in information dependent acquisition mode, which consisted of a high resolution TOF-MS survey scan followed by 20 MS/MS in a second with a maximum accumulation time of 50 ms. First stage MS analysis was performed in positive ion mode over the mass range *m/z* 350–1800 with a 0.5 s accumulation time. The ionspray voltage was set to 2500 V, the curtain gas was set to 25, the nebuliser gas to 12 and the heated interface was set to 150°C. Tandem mass spectra were acquired over the mass range *m/z* 80–1800 using rolling collision energy for optimum peptide fragmentation. Precursor ion masses were excluded for 6 s after two occurrences.

Tandem mass spectrometry data was searched against *in silico* tryptic digests of Poaceae proteins of the Uniprot (version 2012/07) database using ProteinPilot™ 4.0.0.0 software (AB/Sciex) with the Paragon Algorithm. All search parameters were defined as iodoacetamide modified with cysteine alkylation, with trypsin as the digestion enzyme. Modifications were set to the “generic workup” and “biological” modification sets provided with this software package, which consisted of 126 possible modifications, for example, acetylation, methylation and phosphorylation. The generic workup modifications set contains 51 potential modifications that may occur as a result of sample handling, for example, oxidation, dehydration and deamidation.

Peptides identified in discovery phase experiments (information dependent acquisition mode above) that mapped to wheat gluten (glutenin and gliadins) proteins with 95% confidence were used for MRM development. The MRM method offered the advantage of measuring three peptide MRM transitions enabling simultaneous relative quantification as well as confirming the identity of the peptides. MRM transitions were determined for each peptide where the precursor ion (Q1) *m/z* was based on the size and expected charge and the fragment ion (Q3) *m/z* values were selected using the data previously collected. The best three transitions were used and the top two MRM transitions were selected per peptide for quantification. The third most intense transition was used as a qualifier.

Scheduled MRM experiments were performed on a 4000 QTRAP mass spectrometer (AB/Sciex) equipped with a TurboV ionization source operated in positive ion mode. Duplicate samples from each beer bottle were chromatographically separated on a Shimadzu Nexera UHPLC (Shimadzu) using a Phenomenex Kinetex C18 (2.1 mm×10 cm) column with a linear gradient of 5–45% acetonitrile over 15 min with a flow rate of 400 µL/min. The eluent from the HPLC was directly coupled to the mass spectrometer. Data were acquired and processed using Analyst 1.5 software™. The scan speed was set to 1000 Da/s and peptides were fragmented in the collision cell with nitrogen gas using rolling collision energy dependent on the size and charge of the precursor ion and a 15 ms dwell time.

### Statistical analysis

Mean MS areas or ELISA readings were transformed as needed to achieve homogenous variance, via either log10(1+concentration) or log10(concentration) as discussed in legends of Table 1, 2 and Tables S3 and S4 in Information S1. In each case significant differences were determined using a one way analysis of variance of the transformed means (ANOVA; GenStat [48]).

## Supporting Information

Information S1 **Supplementary Results and Methods, Figures and Tables.** **Table S1:** Total protein content of flour, malt wort and beer from test grains. **Table S2:** Peptides used for MRM Quantification. **Table S3:** ANOVA analysis of (log10 ELISA) transformed content of gluten free and low gluten beers. **Table S4:** ANOVA analysis of (log10 hordein +1) transformed peptide content of beers by MS. **Figure S1:** Western blot of 8.3 µg of total protein per lane visualised with 1 in 2000 diluted rabbit polyclonal anti-serpin Z4 antibody, followed by 1/2000 diluted donkey-anti-rabbit from flour, malt, wort, and beer produced from: cv Sloop (A); Risø 56 (B); Risø 1508 (C); and ULG 2.0 (D). Serpin Z4 is the dominant band seen at approximately 43 kDa in each blot (*1), the triple bands seen in flour samples are presumably native serpin Z4 isoforms (*1a, *1b, *1c). Pre-stained molecular weight markers (M, Invitrogen), were calibrated against a 10 kDa unstained protein ladder (Invitrogen) and used to determine the migration of protein bands on the western blot. **Figure S2.** Western blot of 8.3 µg of total protein pre lane visualised with 1/2000 diluted rabbit polyclonal anti-LTP1 antibody, followed by 1/2000 diluted donkey-anti-rabbit from flour, malt, wort, and beer produced from: cv Sloop (A); Risø 56 (B); Risø 1508 (C); and ULG 2.0 (D). The faint band at 43 kDa (*1) was due to pre-existing serpin Z4 conjugate that was not completely denatured during the membrane stripping. The dominant band seen at 9 kDa was due to LTP (2_*_). The faint band seen in the flour samples at 9 kDa was due to native LTP. Pre-stained molecular weight markers (M, Invitrogen), were calibrated against a 10 kDa unstained protein ladder (Invitrogen) and used to determine the migration of protein bands on the western blot. **Figure S3.** A typical standard curve of total Sloop hordeins generated using the ELISA systems kit showing the A450 vs hordein content in µg/kg (ppb). The A450 is shown for triplicate hordein concentrations. A sigmoidal curve of best fit was calculated using GraphPAD Prism 5.04 with an r^2^ of 0.9685. **Figure S4.** The lack of correlation between ELISA values for 60 beers and the relative level determined by MS for avenin (A), B1-hordein (B), B3-hordein (C), D-hordein (D), γ3-hordein (E), or the sum of all relative levels of peptides determined by MS (F), using GraphPAD Prism 5.04. No figures had an R^2^ value greater than 0.06 confirming a lack of any meaningful correlation. **Figure S5.** Comparison of the response of the two D-hordein derived tryptic peptides across the range of beers tested. The values represent the average determined for two individual beer bottles; for clarity the error bars have been omitted however the variation between the bottles was <12% for all gluten-containing beers. The peptide common to wheat showed an increase in the percentage peak area (relative to average) when compared to the peptide unique to barley in beers 5, 6, 9, 16, 30, 45, 55 and 56 (all wheat beers). **Figure S6.** The effect of lyophilisation and dissolution of wort and beer in Urea/DTT, compared with direct sampling on SDS-PAGE. Solutions from cv Sloop (Sloop) or Risø 56 (Risø 56), wort (W) or beer (B) were either sampled directly, the protein measured and 20 µg added directly to Urea/SDS (D), or lyophilised, redissolved in Urea/DTT, the protein measured and 20 µg added to Urea/SDS (Ly). In each case the intensity of protein bands after SDS-PAGE was more intense in the solutions which had been lyophilised, indicating that this treatment was more successful in denaturing proteins, and produced a higher concentration of homogenous protein bands which ran as a coherent band during SDS-PAGE.
(DOC)
