# Supplementary material for: Measuring Hordein (Gluten) in Beer – A Comparison of ELISA and Mass Spectrometry
Source: PLoS One. 2013 Feb 28;8(2):e56452. doi: 10.1371/journal.pone.0056452 (PMC3585340; doi:10.1371/journal.pone.0056452)
Supplement: Information S1 — Supplementary Results and Methods, Figures and Tables. Table S1: Total protein content of flour, malt wort and beer from test grains. Table S2: Peptides used for MRM Quantification. Table S3: ANOVA analysis of (log10 ELISA) transformed content of gluten free and low gluten beers. Table S4: ANOVA analysis of (log10 hordein +1) transformed peptide content of beers by MS. Figure S1: Western blot of 8.3 µg of total protein per lane visualised with 1 in 2000 diluted rabbit polyclonal anti-serpin Z4 antibody, followed by 1/2000 diluted donkey-anti-rabbit from flour, malt, wort, and beer produced from: cv Sloop (A); Risø 56 (B); Risø 1508 (C); and ULG 2.0 (D). Serpin Z4 is the dominant band seen at approximately 43 kDa in each blot (*1), the triple bands seen in flour samples are presumably native serpin Z4 isoforms (*1a, *1b, *1c). Pre-stained molecular weight markers (M, Invitrogen), were calibrated against a 10 kDa unstained protein ladder (Invitrogen) and used to determine the migration of protein bands on the western blot. Figure S2. Western blot of 8.3 µg of total protein pre lane visualised with 1/2000 diluted rabbit polyclonal anti-LTP1 antibody, followed by 1/2000 diluted donkey-anti-rabbit from flour, malt, wort, and beer produced from: cv Sloop (A); Risø 56 (B); Risø 1508 (C); and ULG 2.0 (D). The faint band at 43 kDa (*1) was due to pre-existing serpin Z4 conjugate that was not completely denatured during the membrane stripping. The dominant band seen at 9 kDa was due to LTP (2*). The faint band seen in the flour samples at 9 kDa was due to native LTP. Pre-stained molecular weight markers (M, Invitrogen), were calibrated against a 10 kDa unstained protein ladder (Invitrogen) and used to determine the migration of protein bands on the western blot. Figure S3. A typical standard curve of total Sloop hordeins generated using the ELISA systems kit showing the A450 vs hordein content in µg/kg (ppb). The A450 is shown for triplicate hordein concentrations. A sigmoida [file pone.0056452.s001.doc]

**Supplementary results:**

***Enrichment of Serpin Z4 and LTP in wort and beer:***

Serpin Z4 was identified as a single band running at approximately 43 kDa in all panels of the anti-serpin Z4 western blot (Fig. S1). There was a dramatic enrichment of the 43 kDa band in wort, when aliquots containing equivalent loadings protein from flour, malt, wort and beer from cv Sloop were compared (Fig. S1A). Relative to the level in flour, the enrichment varied from in excess of 3.6 fold (cv Sloop) to 25.3 fold (RisØ 1508). This pattern was similar for all grain samples tested, with extracts of RisØ 1508 and ULG 2.0 (Fig. S1C & D respectively) containing relatively less serpin Z4 than cv Sloop. The faint band at 91 kDa appears to be an artefactual dimer of serpin Z4. The western band at approximately 43 kDa in extracts of flour corresponds to native forms of serpin Z4 (Fig. S1A). The presence of other western bands at 47 and 50 kDa in flour extracts was unexpected and has not been previously reported. The proportion of these higher molecular weight bands also differed with other cultivars.

LTP1 was detected as the dominant band at approximately 9 kDa in western blots from flour, malt, wort, and beer produced from cv Sloop, RisØ 56, RisØ 1508 and ULG 2.0 (Fig. S2; A, B, C, & D respectively). Native LTP1 isoforms were seen as faint bands at 9 kDa in extracts of flour. The LTP proteins were also dramatically enriched in wort, as seen with serpin Z4.

**Supplementary methods:**

***Preparation of anti-LTP and anti-Z4 antibodies.***

Rabbit anti-peptide antibodies to LTP1 and serpin Z4 were produced by Genscript (Piscataway, USA) as follows: antigenic peptides with calculated antigenicity based on Jameson & Wolf algorithm >0.9 [47] were identified within LTP1 (P07597.1; D_33_LHNQAQSSGDRQT_46_) and serpin Z4 (P06293.2; R_258_LSTEPEFIENHIP_271_). The peptides were synthesised with N-terminal cysteines and were amidated at the C-terminus and shown to be 96.3% and 87.9% pure respectively by reverse phase HPLC. Peptides were chosen to minimise lysine content and therefore reduce glycation of the protein during malting and mashing, which could interfere with the antibody binding to the protein. MS confirmed the expected *m/z* for both peptides. The peptides were conjugated to KLH via the N-terminal cysteine and antibodies raised in duplicate rabbits by four immunisations with the conjugate. Antibodies were purified by peptide affinity chromatography, lyophilised and dissolved at 1 mg/mL in PBS containing 0.02% (w/v) azide and the antibody preparation with the highest titre selected (LTP: Rabbit V6177; Serpin Z4: Rabbit V6175 both had titres > 128,000). The titre was the highest dilution that gave an ELISA reading Sample/Blank >2.1 by ELISA. ELISA wells were coated with 100 µL of 4 µg/mL free peptide in PBS and interrogated with Goat Anti-Rabbit IgG (H+L), HRP conjugate.

**Supplementary Tables:**

**Table S1:**

| Cultivar | Sample | Mean ± S.E.^a^ |
| --- | --- | --- |
| Sloop | Flour | 129.0 ± 3.7 |
|  | Malt | 90.0 ± 0.9 |
|  | Wort | 3.5 ± 0.02 |
|  | Beer | 2.8 ± 0.02 |
|  | | |
| RisØ 56 | Flour | 136.4 ± 5.8 |
|  | Malt | 112.5 ± 6.3 |
|  | Wort | 3.5 ± 0.09 |
|  | Beer | 2.2 ± 0.08 |
|  | | |
| RisØ 1508 | Flour | 154 ± 3.3 |
|  | Malt | 104.0 ± 3.1 |
|  | Wort | 2.5 ± 0.04 |
|  | Beer | 1.6 ± 0.01 |
|  | | |
| ULG 2.0 | Flour | 153 ± 2.3 |
|  | Malt | 127 ± 2.3 |
|  | Wort | 3.3 ± 0.01 |
|  | Beer | 0.69 ± 0.05 |

^a^ The total protein content of flour and malt is given as the mean ± S.E. (n=3) as mg protein/ mg flour (flour and malt), or mg protein/mL solution (wort and beer).

**Table S2:** Peptides used for MRM Quantification

| Gluten Family | Protein Accession | Peptide Sequence^a^ | Species | Unique |
| --- | --- | --- | --- | --- |
| Avenin | F2EGD5 | QQCCQPLAQISEQAR | TriticumAegilopsHordeum | 🗶 |
| B1-hordein | Q40020 | VFLQQQCSPVR | Hordeum | ✓ |
| B3-hordein | Q4G3S1 | VFLQQQCSPVPMPQR | Hordeum | ✓ |
| D-hordein | Q84LE9 | ELQESSLEACR | TriticumHordeumAegilopsSecale | 🗶 |
| D-hordein | Q84LE9 | QYEQQTEVPSK | Hordeum | ✓ |
| γ3-hordein | P80198 | QQCCQQLANINEQSR | Hordeum | ✓ |
| α-gliadin | Q9M4L6 | (p)QQILQQQLIPCR | TriticumAegilopsSecale | 🗶 |
| α-gliadin | Q9M4L6 | VPVPQLQPQNPSQQQPQEQVPL | TriticumAegilopsSecale | 🗶 |
| HMW-glutenin | P10388 | IFWGIPALLK | TriticumAegilops | ✓ |
| LMW-glutenin | B2BZC7 | SIVLQEQQQVR | Triticum | ✓ |

^a^(p) refers to pyroglutamic acid N-terminal modification.

**Table S3**:

| Beer | 17 | 47 | 49 | 50 | 51 | 52 | 57 | 58 | 59 | 60 | LSD |
| --- | --- | --- | --- | --- | --- | --- | --- | --- | --- | --- | --- |
| Mean Log10 ELISA | -0.855a | -0.067b | 0.291c | -1.043a | -0.965a | -1.254a | -1.162a | -1.136a | -1.187a | -1.175a | 0.3409 |

The mean of triplicate ELISA readings for two replicate bottles for each beer, were log transformed and averaged and the means compared by one way ANOVA for each peptide class. Transformed means with the same letter within each row were not significantly different at the 5% level. The LSD for pairwise comparison at 5% level was 0.3409; omission of beer 17 gave a lower LSD of 0.138.

**Table S4**:

| Beer | 17 | 47 | 49 | 50 | 51 | 52 | 57 | 58 | 59 | 60 | LSD |
| --- | --- | --- | --- | --- | --- | --- | --- | --- | --- | --- | --- |
| Avenin | 0.046a | 0.694c | 0.334b | 0.068a | 0.210a | 0.022a | 1.595d | 0.066a | 2.103e | 0.000a | 0.1654 |
| B1 | 0.179a | 0.319a | 0.321a | 0.124a | 0.307a | 0.281a | 2.367c | 0.519a | 1.795b | 0.412a | 0.3737 |
| B3 | 0.146a | 0.588b | 0.377ab | 0.185a | 0.157a | 0.208a | 0.496b | 0.160a | 0.862b | 0.320a | 0.3035 |
| D | 0.064a | 0.531b | 0.108a | 0.088a | 0.086a | 0.011a | 1.888d | 0.185a | 1.625c | 0.113a | 0.1463 |

The mean of four replicates of the mean peptide content by MS, were log transformed and analysed by one way ANOVA for each peptide class. Transformed means with the same letter within each row were not significantly different at the 5% level. The LSD for pairwise comparison is shown for each row.

**Supplementary Figures.**

**Figure S1.**

**Figure S2.**

**Figure S3.**

**Figure S4.**

**
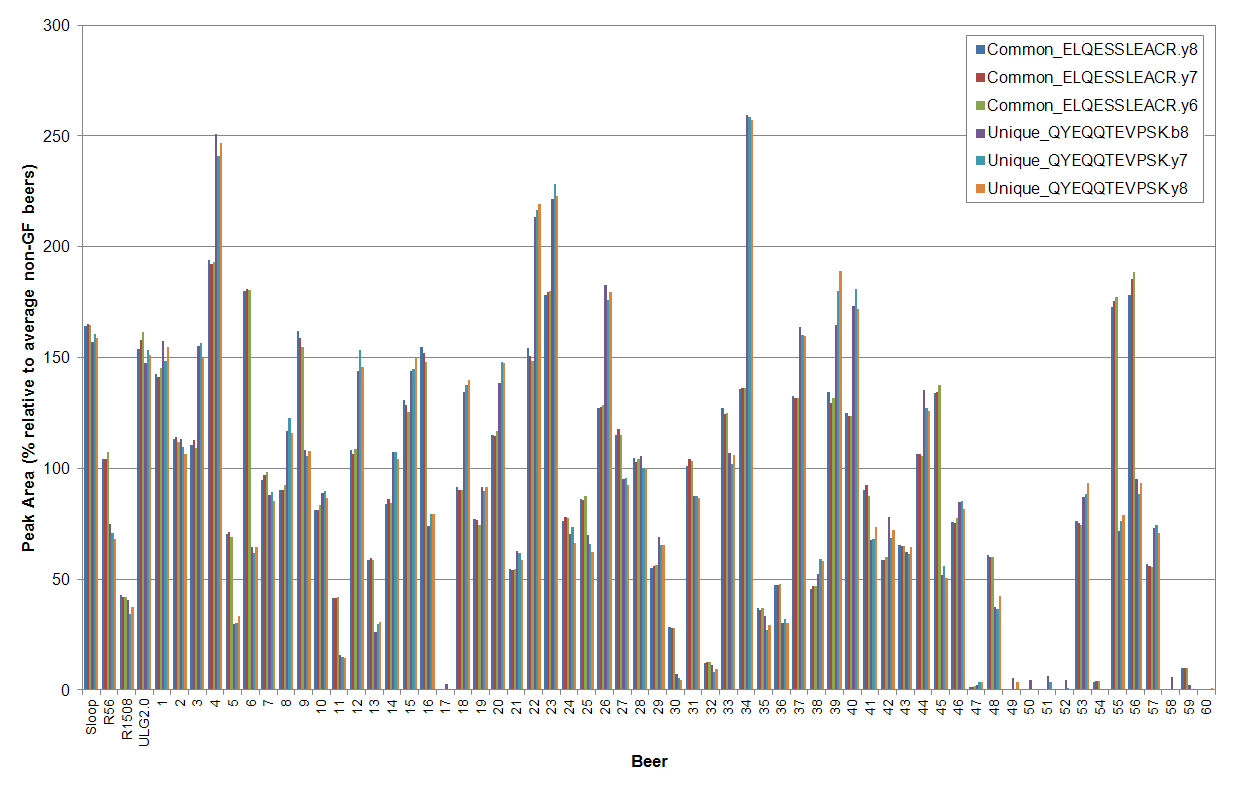
**

**Figure S5.**

**Figure S6.**
